# Unveiling Corrosion Pathways of Sn Nanocrystals through High-Resolution Liquid Cell Electron Microscopy

**DOI:** 10.1021/acs.nanolett.3c03913

**Published:** 2024-01-22

**Authors:** Xinxing Peng, Junyi Shangguan, Qiubo Zhang, Matthew Hauwiller, Haobo Yu, Yifan Nie, Karen C. Bustillo, A. Paul Alivisatos, Mark Asta, Haimei Zheng

**Affiliations:** † Materials Science Division, Lawrence Berkeley National Laboratory, Berkeley, California 94720, United States; ‡ Department of Materials Science and Engineering, University of California, Berkeley, Berkeley, California 94720, United States; § Department of Chemistry, University of California, Berkeley, Berkeley, California 94720, United States; ∥ Beijing Key Laboratory of Failure, Corrosion and Protection of Oil/Gas Facility Materials, College of New Energy and Materials, China University of Petroleum, Beijing, Beijing 102249, China; ⊥ National Center for Electron Microscopy, Molecular Foundry, Lawrence Berkeley National Laboratory, Berkeley, California 94720, United States; # Kavli Energy NanoScience Institute, University of California, Berkeley, and Lawrence Berkeley National Laboratory, Berkeley, California 94720, United States

**Keywords:** liquid cell TEM, Sn nanocrystal, pitting corrosion, uniform corrosion

## Abstract

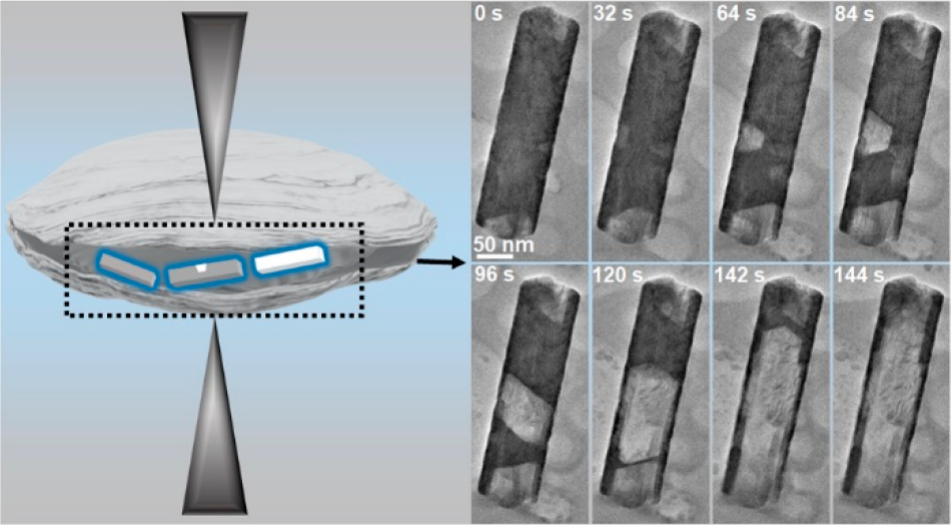

Unveiling materials’ corrosion pathways is significant for understanding the corrosion mechanisms and designing corrosion-resistant materials. Here, we investigate the corrosion behavior of Sn@Ni_3_Sn_4_ and Sn nanocrystals in an aqueous solution in real time by using high-resolution liquid cell transmission electron microscopy. Our direct observation reveals an unprecedented level of detail on the corrosion of Sn metal with/without a coating of Ni_3_Sn_4_ at the nanometric and atomic levels. The Sn@Ni_3_Sn_4_ nanocrystals exhibit “pitting corrosion”, which is initiated at the defect sites in the Ni_3_Sn_4_ protective layer. The early stage isotropic etching transforms into facet-dependent etching, resulting in a cavity terminated with low-index facets. The Sn nanocrystals under fast etching kinetics show uniform corrosion, and smooth surfaces are obtained. Sn nanocrystals show “creeping-like” etching behavior and rough surfaces. This study provides critical insights into the impacts of coating, defects, and ion diffusion on corrosion kinetics and the resulting morphologies.

Corrosion is often a major concern for the applications of metals and alloys. It may lead to structural damage and failure of devices,^1,2^ for example, fuel cell breakdown due to electrode degradation,^3−5^ performance loss of solar cells due to etching of silicon,^6^ deterioration of electronic packaging,^7^ and so on. Corrosion of structural materials can also impact the safety of workers and induce significant economic loss.^8−11^

Materials corrosion has been a topic with extensive studies.^12,13^ Strategies have been developed to improve materials corrosion resistance.^14,15^ However, even with protective measures, such as applying a thin film coating of corrosion-resistant materials, corrosion-induced material degradation has still been frequently reported.^16^ In these materials with surface coating, pitting corrosion is most common, where cavities are produced.^17^ Compared to uniform corrosion, pitting corrosion can be more difficult to detect, as corrosion products may cover the pits. And, pitting corrosion is considered hard to predict or prevent.

Corrosion of metals and alloys may arise from oxidation or galvanic reactions.^18^ It has been traditionally investigated by spectroscopic measurements, such as electrochemical impedance spectroscopy, cyclic or potentiodynamic polarization experiments, or surface inspection.^19,20^ Based on the measured corrosion kinetics and by inspecting the materials’ surfaces before and after, valuable information has been obtained and theories have been developed.^21^ However, it has been a challenge to reveal the corrosion pathways at the nanoscale due to the lack of the capability of direct observation with high spatial and temporal resolution; thus, it limits the understanding of corrosion mechanisms. Corrosion research should be performed at the nanolevel and even atomic level to gain a clearer and more fundamental picture of corrosion behaviors, like pitting, which starts at an extreme small scale down to nanometers and even to atomic scale.^22^

Liquid cell transmission electron microscopy (TEM) provides the opportunity to observe nanoscale material transformations in liquids in real time.^23^ With the recent advances in liquid cell design and nanofabrication, and employing the aberration-corrected electron microscope, state-of-art electron detection, and image analysis, liquid cell TEM has been widely used to directly observe various materials dynamic phenomena in solution with high spatial resolution.^24^ For example, the nucleation and growth of nanocrystals,^25^ solid electrolyte interphases during the electrodeposition of alkali metal,^26,27^ structure and bonding of liquids,^28^ short-range ordering in liquid electrolyte,^29^ and many other topics have been studied. Liquid cell TEM has also been used to study nanocrystal degradation,^30^ etching, and galvanic reactions of metal.^2,16,31−37^ Thus, with an innovative design of experiments, it is possible to reveal corrosion pathways of materials at the nanoscale or at the atomic level using the powerful liquid cell TEM platform.

In this work, using liquid cell TEM we investigated the oxidative etching of Sn nanocrystals with or without a surface protection layer of Ni_3_Sn_4_. Sn@Ni_3_Sn_4_ nanocrystals are an ideal model system for the study of pitting corrosion at the nanoscale and a valuable counterpart for the study of uniform corrosion. The alloy surface layer is more corrosion-resistant than Sn;^38^ thus, it resembles a protective thin film coating on bulk metal. To trace the structural, morphological, and chemical changes during the corrosion of Sn nanocrystals, we employed thin carbon film liquid cells. These liquid cells enable atomic resolution imaging, as well as “freeze-and-look” by cryogenic electron microscopy (Cryo-EM), and electron energy dispersive X-ray spectroscopy (EDS) with a large collection angle. We focus on the nanocrystal surfaces and solid–liquid interfaces in order to elucidate the initiation and progression of pitting corrosion of Sn@Ni_3_Sn_4_ nanocrystals and to compare it with the uniform corrosion of Sn nanocrystals without coating. An unprecedented level of information at the atomic level has been achieved, which provides valuable insights into corrosion behavior beyond the nanoscale.

We have developed thin carbon film liquid cells (Figure 1a) for an in situ TEM study of the corrosion behavior of Sn with or without a protective layer of Ni_3_Sn_4_ in an aqueous solution of salt chlorides (e.g., including SnCl_4_, NiCl_2_, and BeCl_2_). To prepare a liquid cell, the carbon-film-supported TEM grids were first treated with oxygen plasma to increase surface hydrophilicity (see the contact angle measurements in Figure S1). The Sn nanorods with/without a protective layer of Ni_3_Sn_4_ are prepared through chemical reactions of Be metal with an aqueous solution of SnCl_4_ with/without NiCl_2_, and the resulting Sn or Sn@Ni_3_Sn_4_ nanocrystals are encapsulated into a liquid cell (Figure S2; also see the Materials and Methods section for details). Then, the liquid cell is loaded into an aberration-corrected TEM for in situ studies. A high-angle annular dark-field scanning transmission electron microscopy (HAADF-STEM) image (Figure 1b) shows the as-synthesized Sn nanorods in a liquid cell. Figure 1c displays a pristine Sn@Ni_3_Sn_4_ nanocrystal. The EDS elemental maps show the spatial distribution of Sn and Ni, where the core is Sn and Ni is abundant at the surface corresponding to Ni_3_Sn_4_ (see further characterization in Figure 1f,g). Elemental maps of chloride (Cl) and oxygen (O) from the bulk solution are also obtained (Figure S3).

**Figure 1:**
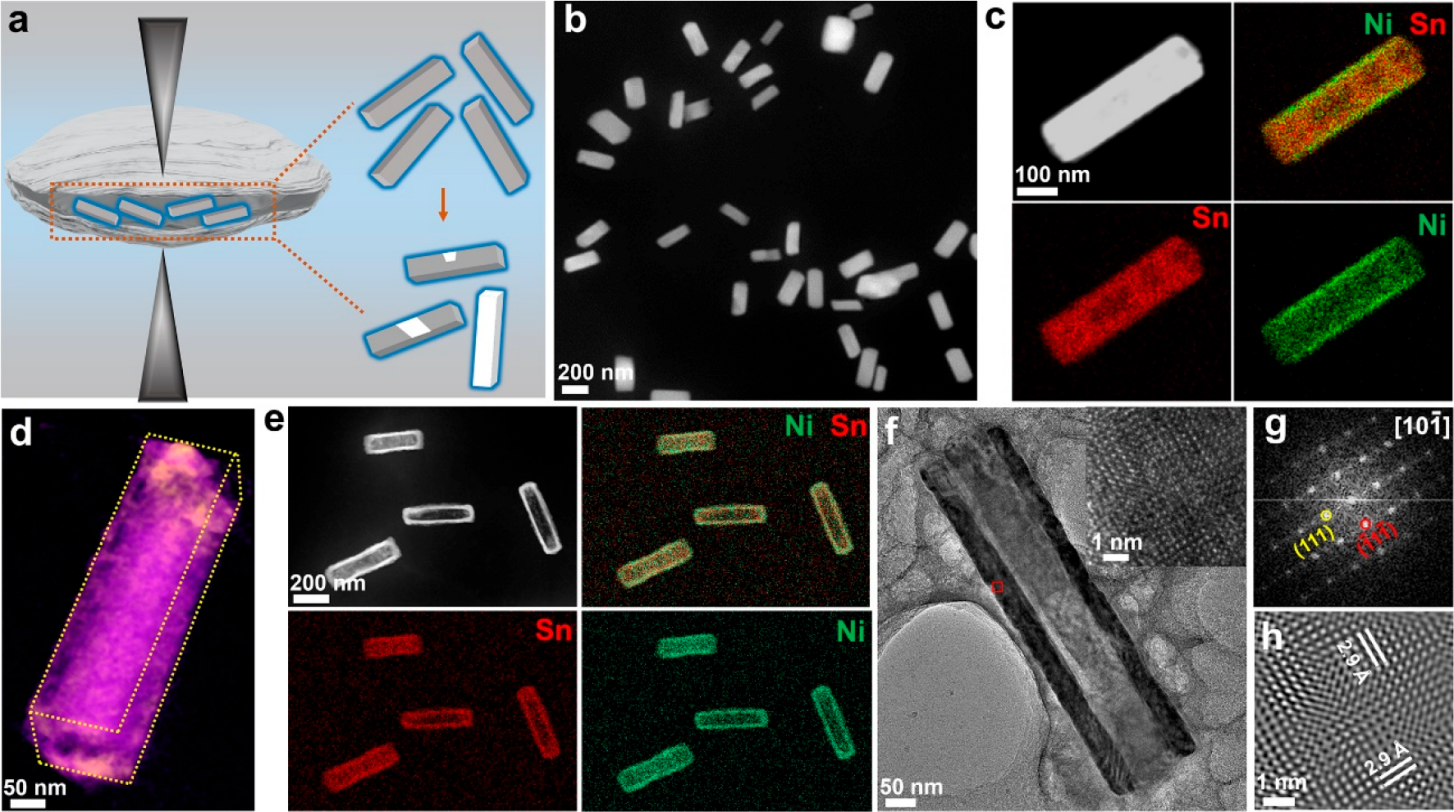
Sn@Ni_3_Sn_4_ nanocrystals before and after corrosion in an aqueous solution characterization under the cryogenic temperature. (a) Schematic illustration of liquid cell TEM experiment. (b) High-angle annular dark-field (HAADF)-STEM images and (c) (energy-dispersive X-ray spectroscopy) EDS elemental mapping of Sn@Ni_3_Sn_4_ nanocrystal in solution before etching. (d) 3D reconstruction of a hollow nanocrystal after etching. The morphology of as-synthesized Sn@Ni_3_Sn_4_ nanocrystal shows a rectangular prism. (e) HAADF-STEM image and energy EDS chemical maps of representative hollow nanocrystals. (f) Low magnification image of a hollow nanocrystal. The inset image displays a magnified view of the region within the red square. (g) Fast fourier Transform (FFT) and (h) inverse FFT image from the area highlighted by the red square in (f).

The Sn nanocrystals, in both cases with and without a protective layer, experience corrosion in a liquid cell during imaging. We first show Sn@Ni_3_Sn_4_ nanocrystals after Sn is completely removed, which results in a hollow nanostructure. To achieve a three-dimensional (3D) view, a set of TEM images of a hollow nanocrystal at different tilting angles were collected (Figure S4). The reconstructed electron tomography reveals a rectangular prism, as shown in Figure 1d (Video S1). Figure 1e shows an HAADF-STEM image and the corresponding EDS elemental maps of the hollow nanostructure, which indicate that the hollow nanocrystal consists of Sn and Ni in the shell. Both the electron tomography experiments and the STEM-EDS of the hollow nanostructures are performed by quickly freezing the liquid cell samples to the cryogenic temperature.

We analyze the crystal structure of a representative hollow nanocrystal (Figure 1f). The fast Fourier transform (FFT) pattern (Figure 1g) from the selected area in Figure 1f and the corresponding high-resolution TEM image (Figure 1h) show that it is consistent with Ni_3_Sn_4_ (space group *C*12/*m*1) viewed along the [10–1] direction.^39^ The lattice *d* spacing marked in Figure 1h matches the (111) and (−11–1) lattice planes of Ni_3_Sn_4_. The as-synthesized Sn@Ni_3_Sn_4_ nanocrystals are core–shell prisms. The Sn core can be completely etched away, leaving the corrosion-resistant Ni_3_Sn_4_ hollow nanostructure.

Real-time observation of the corrosion of Sn@Ni_3_Sn_4_ nanocrystals using liquid cell TEM captures the morphological evolution (see Figure 2a and Video S2). An electron dose rate of 1210 e^–^ Å^–2^ s^–1^ is maintained during imaging. Based on the contrast changes, the Sn core is etched away in multiple locations within the Sn@Ni_3_Sn_4_ nanocrystal. As etching proceeds, cavities with a trapezoidal cross section are obtained (Figure 2a). Multiple cavities can connect to form a large cavity. Eventually, the Sn core completely disappeared, leaving a Ni_3_Sn_4_ hollow nanocrystal.

**Figure 2:**
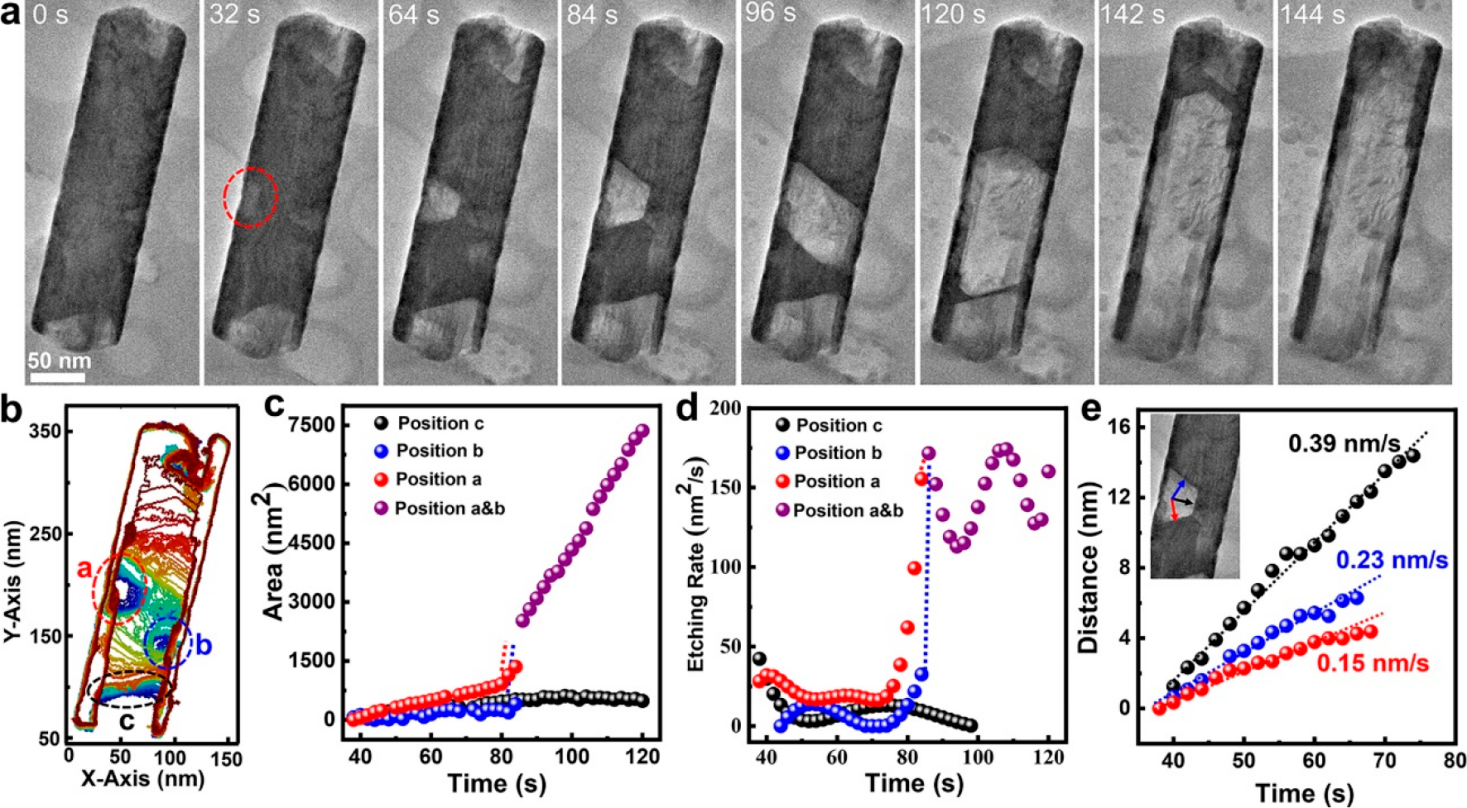
Real-time observation of the etching of a Sn@Ni_3_Sn_4_ nanocrystal in an aqueous solution. (a) Sequential TEM images showing the etching process at a dose rate of 1210 e^–^ Å^–2^ s^–1^. (b) Contours of the Sn@Ni_3_Sn_4_ nanocrystal during etching. The color shows a time sequence with blue as the initial time and red as the later time. (c) Etching area and (d) etching rate evolutions of the cavities (a, b, and c) as highlighted in (b). (e) From the plots of “etching distance versus time” along the highlighted directions (see the inset), the etching rates of different facets are calculated.

We trace the contours of each cavity during etching, and the contour plots are shown in Figure 2b with different colors corresponding to the time evolution (blue as the initial time and red as the end). We compare the etching rate at three reaction sites (marked as **a**, **b**, and **c** in Figure 2b), by measuring the projected area of each cavity with time (Figure 2c). Positions **a** and **b** represent the reaction sites with a protective layer of Ni_3_Sn_4_ (an average thickness of ∼12 nm in the local region), and position **c** at the end of the nanorod is without an obvious protective layer. The two cavities marked with **a** and **b** coalesce to form one large cavity at a later stage (at 84 s). The etching rates at the three reaction sites and the merged regions of the **a** and **b** cavities are plotted in Figure 2d. Remarkably, roughly similar rates of a, b and c cavities are obtained. However, a significantly higher etching rate (about 13 times) is found after the a and b cavities are merged.

In addition, we measured the etching rate along each individual facet of one representative cavity. As highlighted with the black, blue, and red arrows in the cavity with trapezoidal cross section, the etching rates of the corresponding facets of (020), (011), and (01–1) are calculated: 0.39, 0.23, and 0.15 nm/s, respectively (Figures 2e and S5). The cavity is terminated with facets {011} after 96 s; these facets have lower corrosion rates.

To reveal the initiation and evolution of a cavity in pitting corrosion, we trace the evolution of a Sn nanocrystal with a thin Ni_3_Sn_4_ shell (∼2 nm) at the atomic scale. Sequential TEM images show that a small semicircular cavity is formed at the beginning, and it subsequently develops into a large faceted cavity, marked as “A” in Figure 3a (also see Video S3 and Video S4). For the TEM images displayed in Figure 3a, a band-pass filter is applied to the original images to improve the signal-to-noise ratio (see Figure S6 for the raw images).

**Figure 3:**
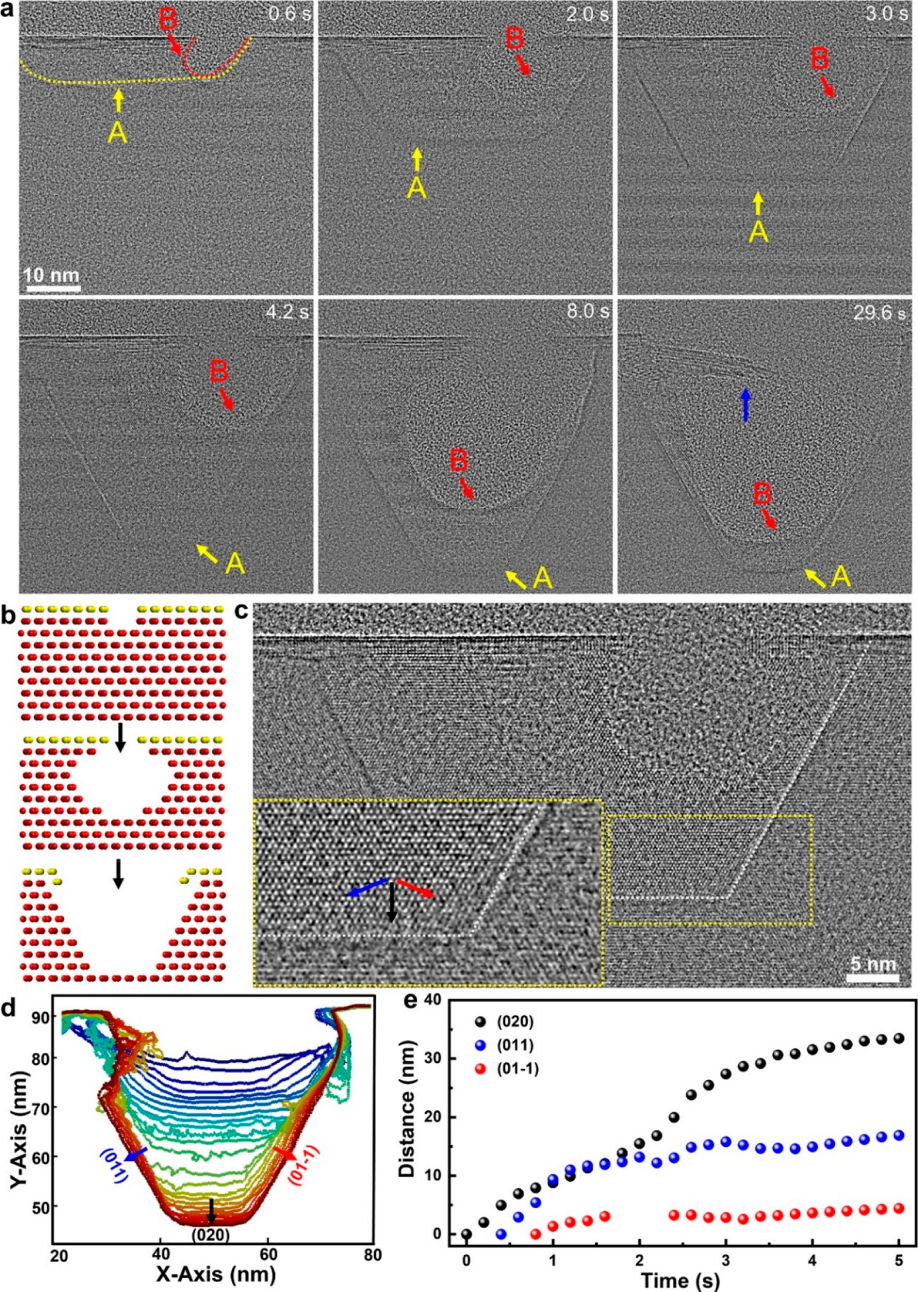
High-resolution real-time observation of the faceted cavity development during pitting corrosion of Sn@Ni_3_Sn_4_ nanocrystal. (a) Sequential TEM imaging showing the faceted cavity development under a dose rate of 6105 e^–^ Å^–2^ s^–1^. (b) Schematic illustration of the cavity development from the initial round to faceted at the later stage. The golden layer represents the protection layer, and the red model represents the Sn metal. (c) Representative high-resolution TEM image showing the cavity with distinct facets. The inset is the close-up view of the selected region. Black, blue, and red arrows show etching direction along (020), (011), and (01–1), respectively. (d) Contours of the cavity during pitting corrosion. The color shows a time sequence with blue as the initial time and red as the later time. (e) Etching rate of different facets.

We find that the pitting corrosion starts from a defective site where the Ni_3_Sn_4_ protective layer is broken. The cavity grows larger as more Sn is removed, during which time the defective region in the Ni_3_Sn_4_ layer maintains the same size. At the early stage, the etching of the Sn metal core is mostly along the interfaces with Ni_3_Sn_4_, leading to a shallow cavity with smooth solid–liquid (Sn–aqueous solution) interfaces. As etching proceeds, more fresh Sn metal is exposed to the solution. Then, the etching of Sn–liquid interfaces becomes facet-dependent. The protective layer collapses eventually, as indicated by the blue arrow at 29.6 s in Figure 3a.

As illustrated in Figure 3b, the pitting corrosion includes two stages: isotropic etching at the early stage and facet-dependent etching at the later stage. A 3D model of the faceted cavity with a trapezoidal cross section is displayed in Figure S7. High-resolution TEM images show the facets of the cavity are (020), (011), and (01–1) planes, as indicated by the black, blue, and red arrows (Figure 3c). The contours of the cavity versus time (Figure 3d) show the cavity evolution from isotropic etching to facet-dependent etching. We calculate the etching rate of each individual facet of cavity **A**. As shown in Figure 3e, the etching rate of facets (020) is higher than those of facets (011) and (01–1), which is consistent with our observation at low magnification (Figure 2e).

To verify that defects on the protective surface layer are critical for the initiation of pitting corrosion, we carefully examined many Sn@Ni_3_Sn_4_ nanocrystals. We notice that a dense protective layer of Ni_3_Sn_4_ on the nanocrystal surface prevents the Sn metal core from being etched (Figure S8). It is worth noting that when bubbles are found nearby, corrosion does not lead to a faceted cavity (Figure S9 and Video S5), which may result from a significantly enhanced etching rate by oxygen gas nanobubbles.^40^ Moreover, following the etching of the Sn metal is the dissolution of the protective layer of Ni_3_Sn_4_. The collapse of the protective layer at a late stage (at 29.6 s in Figure 3a) demonstrates that Ni_3_Sn_4_ can be destroyed eventually. Thus, cavity **B** in Figure 3a is assigned as a hole from the Ni_3_Sn_4_ shell being dissolved.

To further understand the corrosion behavior, we investigate the corrosion of Sn metal nanocrystals without a coating using liquid cell TEM. Sn nanocrystals display nanorod and nanocube morphologies, as shown in Figure S10. Cryo-EM is used for STEM-EDS characterization. EDS elemental maps show that they are Sn metal nanocrystals without surface oxidation. As a comparison, we also study Sn nanocrystals synthesized ex situ with an obvious oxide layer after several rounds of centrifugation and washing in the air a liquid cell (Figure S11), which shows different behavior of Sn with an oxide layer.

Liquid domains with different sizes can be found in a carbon thin film liquid cell. Based on their image contrast differences, the larger liquid domains (thicker liquid) can be easily distinguished from the thin liquid area. The liquid thickness of different domains ranges from tens of nanometers to hundreds of nanometers. We focus on the solid–liquid interfaces of Sn nanocrystals during corrosion by comparing those in a thin liquid layer and an abundant solution (an aqueous solution of SnCl_4_, NiCl_2_, and BeCl_2_). Figure 4a (Video S6) shows the corrosion behavior of a Sn nanocrystal in an abundant solution. Fast etching kinetics shows that the whole nanocrystal is completely dissolved within a few seconds. Smooth and round interfaces are found during the etching of the Sn nanocrystal. Accompanied by the dissolution of the primary Sn nanocrystal, small Sn nanoparticles are formed nearby. This suggests that the Sn ion concentration increases rapidly to the level of oversaturation from the etching of the primary Sn nanocrystal.

**Figure 4:**
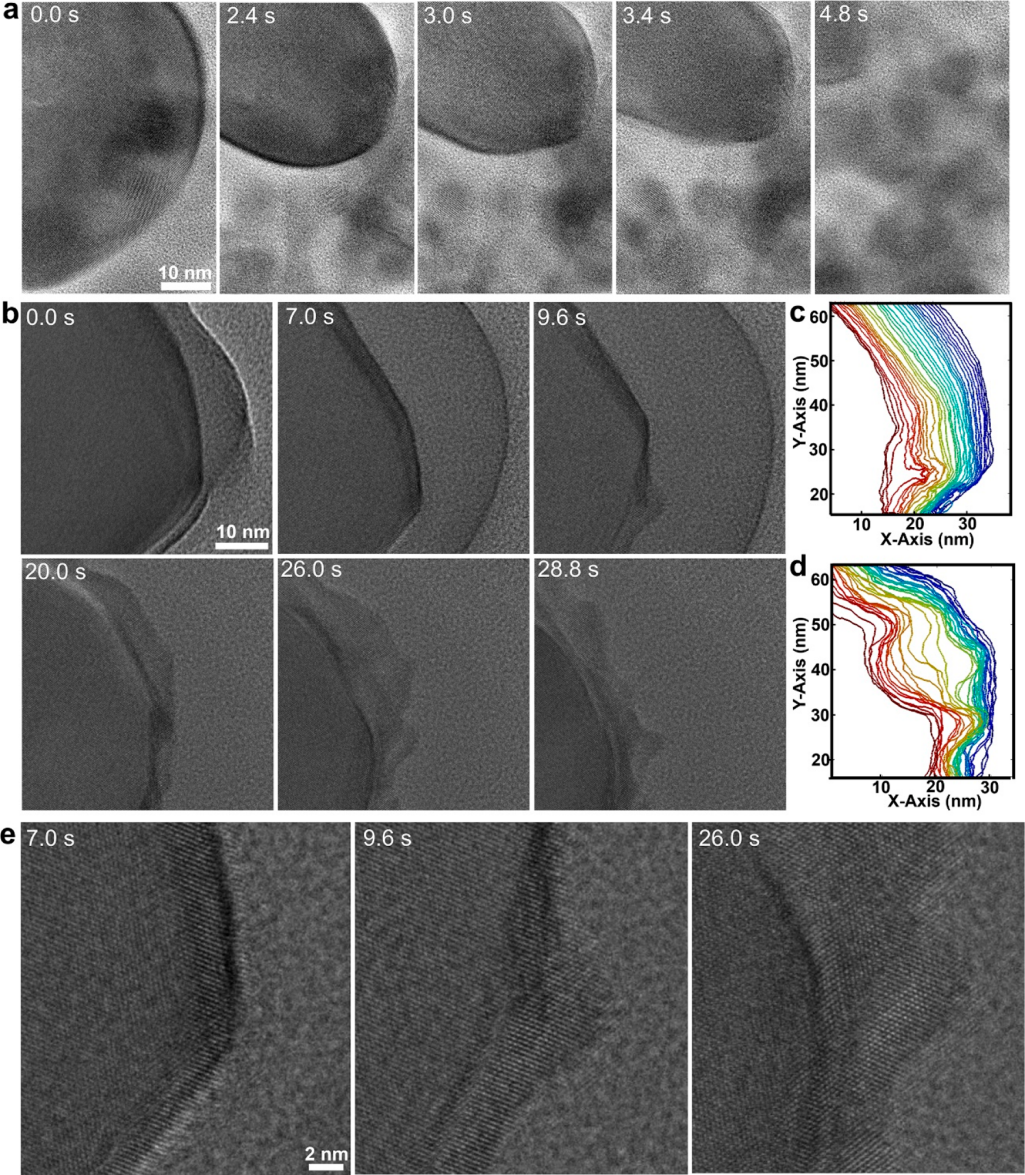
Corrosion of Sn nanocrystals in solution. (a) Sequential TEM images showing the etching of a Sn nanocrystal in thick liquid at a dose rate of 7470 e^–^ Å^–2^ s^–1^. (b) Sequential high-resolution TEM imaging showing corrosion of an Sn nanocrystal in a thin liquid region at a dose rate of 12000 e^–^ Å^–2^ s^–1^. (c) Contours of the Sn nanocrystal surfaces at the beginning (0.0–13.8 s) and (d) late (20.0–33.0 s) during corrosion of the Sn nanocrystal in (b). The colors show a time sequence with blue as the initial time and red as the later time. (e) Enlargement of representative images in panel b.

For a Sn nanocrystal in a thin liquid layer (estimated to be tens of nanometers, as shown in Figure S12), distinctly different corrosion behavior is found. Sequential images (Video S7) show that the Sn nanocrystal surfaces become rough during etching (Figure 4b–e). Then, “creeping-like” etching behavior can be found. As shown in Figure 4b–e, the Sn nanocrystal surfaces display “ocean wave-like” movements. Remarkably, a single crystalline feature remains even at the tips of the curvature (Figure 4e). It takes much longer to have the Sn nanocrystal completely dissolve compared with that in Figure 4a. Additionally, a dense Sn ion solution of the etching products moves away from the Sn nanocrystal surfaces (Figure 4b). The dense layer of Sn ion etching products shows higher contrast compared to the background solution; thus, it can be easily distinguished. We consider the reduced ion diffusion, including Sn ions diffusing away from the Sn nanocrystal and the transport of oxidative species to the Sn nanocrystal surfaces, plays a significant role in the observed slow corrosion kinetics and the distinct solid–liquid interfaces.

For the corrosion of Sn and Sn@ Ni_3_Sn_4_ nanocrystals in an aqueous salt chloride solution (including SnCl_4_, NiCl_2_, and BeCl_2_), the etchant is considered to be Sn^4+^ ions in the solution (Sn^4+^ + 2e^–^ → Sn^2+^). Thus, the redox couple during the corrosion of Sn nanocrystals can be expressed as
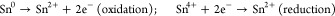
Radiolysis of water molecules under the electron beam can generate a variety of reaction products, including e_h_^–^, H^•^, OH^•^, H_2_, H_2_O_2_, O_2_, HO_2_^•^, etc.^41^ The strong oxidative species, such as OH^•^ and H_2_O_2_, can contribute to the oxidation of Sn metal into Sn ions. In this work, radiolysis of aqueous halide solutions may also produce halogen gases, such as Cl_2_, which may accelerate the etching.^42^ However, it is unlikely to produce an appreciable amount of Cl_2_ gases in our in situ experiments; thus, this effect is negligible.^40^ In addition, the aqueous solution may become more acidic due to the presence of BeCl_2_ salt in the solution,^43^ which can enhance the etching rate of Sn nanocrystals. Nanobubbles have been observed in our experiments (Figure S9), and O_2_ nanobubbles from radiolysis of water by O_2_ nanobubbles may enhance the local etching of Sn metal. In addition, we observed that chloride ions have the potential to accelerate the corrosion reaction by comparing the etching behavior of Sn in aqueous solutions with and without chloride ions (Figure S13).

Our control experiments indicate that the etching rate of Sn increases with the increase in electron beam dose. Thus, a constant electron beam current density is maintained during each liquid cell TEM experiment. Under a consistent electron beam dose, we can compare the different etching rates of different facets during the etching of the Sn core within a Sn@Ni_3_Sn_4_ nanocrystal.

Our in situ liquid cell TEM studies in liquid cells have demonstrated that Sn nanocrystals with or without a protective layer of Ni_3_Sn_4_ experience distinctly different corrosion behavior. We have shown that for the pitting corrosion of Sn@Ni_3_Sn_4_, defects in the protective layer are critical for the initiation of Sn corrosion (Figure 3). Eventually, a cavity with the terminating low-energy facets of (020), (011), and (01–1) is obtained. The slow ion diffusion through the small opening of the cavity likely plays a major role in the facet-dependent etching, during which a pseudoequilibrium state can be achieved. The facet-dependent etching behavior can be reproduced using kinetic Monte Carlo (KMC) simulation (see details in Figure S14 and Video S8). Facets with high corrosion rate will disappear eventually, leaving the cavity terminated with facets with low corrosion rate, such as facets {020} and {011} with lower energy. The reduced reaction kinetics may allow the Sn nanocrystals to reach a pseudoequilibrium state.^44^ The corrosion rate difference between the {020} and {011} facets can be related to the atomic arrangement. Metallic β-Sn has a body-centered tetragonal crystal structure (Figure S15).^45^ The (020) facet corresponds to a plane with a lower atomic packing density compared with the (011) facet. This lower packing density suggests that the atomic bonds in the (020) plane might be weaker or less tightly packed, making it more susceptible to etching in an aggressive environment. The weaker bonds can be more easily broken or attacked by the etching solution, leading to a higher etching rate.

For the “uniform” etching of Sn nanocrystals without a protective Ni_3_Sn_4_ shell, smooth and round surfaces have been achieved due to the fast corrosion kinetics in a thick liquid layer (Figure 4a). For the Sn nanocrystal in a thin liquid layer, our observation of “rough” nanocrystal surfaces is unique (Figure 4b), which likely results from 2D ion diffusion in a thin liquid film. An unseen “creeping-like” etching behavior has been found. Thus, the ability to control ion diffusion at the solid–liquid interfaces, including the transport of oxidative species to the interfaces and the reaction products diffusing away from the interfaces, is key to controlling the morphologies of metal surfaces during etching.

In summary, using in situ liquid cell TEM, we have studied the corrosion behavior of Sn and Sn@Ni_3_Sn_4_ nanocrystals down to the atomic level. Through tracking of the corrosion pathways at the nanoscale and the atomic level, the impacts of defects, surface coating, and ion diffusion on the evolution of solid–liquid interfaces are revealed. This study allows a fundamental understanding of the corrosion mechanisms and sheds light on strategies for tuning and controlling interfaces during the etching of metals.

## Data Availability

All data needed to evaluate the conclusions in the paper are present in the paper and/or the Supporting Information. Additional data related to this paper may be requested from the authors.

## Supporting Information Available

Details for the setup of the liquid-cell experiment, structure and composition characterization of the Sn metal core and alloy shell, electron tomography images and 3D model of the Sn@Ni_3_Sn_4_ nanoparticles, liquid layer thickness calculation, and KMC simulation (PDF)
Video S1: 3D reconstruction of a single nanocrystal (AVI)
Video S2: In situ liquid cell TEM movie showing the pitting corrosion process of Sn@Ni_3_Sn_4_ nanocrystals (AVI)
Video S3: in situ liquid cell TEM movie showing the pitting corrosion process of Sn@Ni_3_Sn_4_ nanocrystals (AVI)
Video S4: in situ liquid cell TEM Video S3 after band-pass filter to show the initial pitting corrosion (AVI)
Video S5: in situ liquid cell TEM movie showing the galvanic corrosion process, and the influence of bubbles on corrosion behaviors at low magnification (AVI)
Video S6: in situ liquid cell TEM movie showing the fast corrosion kinetics of metallic Sn nanocrystals in the thick liquid layer at high magnification (AVI)
Video S7: in situ liquid cell TEM movie showing the “creep-like” etching process and diffusion layer during the uniform corrosion process in the thin liquid layer at high magnification (AVI)
Video S8: kinetic Monte Carlo simulation on the corrosion of Sn with protection layer (AVI)

## Supplementary Material

nl3c03913_si_001.pdf

nl3c03913_si_002.avi

nl3c03913_si_003.avi

nl3c03913_si_004.avi

nl3c03913_si_005.avi

nl3c03913_si_006.avi

nl3c03913_si_007.avi

nl3c03913_si_008.avi

nl3c03913_si_009.avi
