# Supplementary material for: Unveiling Corrosion Pathways of Sn Nanocrystals through High-Resolution Liquid Cell Electron Microscopy
Source: Nano Lett. 2024 Jan 22;24(4):1168–75. doi: 10.1021/acs.nanolett.3c03913 (PMC10835717; doi:10.1021/acs.nanolett.3c03913)
Supplement: Supplementary file 1 — nl3c03913_si_001.pdf [file nl3c03913_si_001.pdf]

Supplementary Materials for

Unveiling corrosion pathways of Sn nanocrystals through high-resolution liquid cell electron microscopy

Xinxing Peng, Junyi Shangguan, Qiubo Zhang, Matthew Hauwiller, Haobo Yu, Yifan Nie, Karen C. Bustillo, A. Paul Alivisatos, Mark Asta and Haimei Zheng**

*Corresponding authors. Email: hmzheng@lbl.gov; pengxinxing0613@gmail.com

This PDF file includes:

Supplementary Text
Materials and Methods
Figure S1. Surface classification based on contact angles.
Figure S2. Schematic of representation of the set-up of the liquid-cell experiment.
Figure S3. Morphology and composition characterization of Sn nanocrystals with nanocoating before and after corrosion.
Figure S4. Electron tomography images of Sn nanocrystals.
Figure S5. Facet determination according to the etched morphology.
Figure S6. High-resolution real-time observation of the oxidative pitting corrosion of $Sn@Ni_3Sn_4$ nanocrystal in aqueous solution.
Figure S7. 3D model of rectangular prism nanocrystal viewing along [100] direction.
Figure S8. Sequential TEM images show that no corrosion reaction occurs where the protective layer is intact.
Figure S9. Sequential TEM images showing a galvanic corrosion process of $Sn@Ni_3Sn_4$ nanocrystal in aqueous solution.
Figure S10. Structural morphology and EDS characterization of Sn nanocrystal within the liquid cell by cryo-EM.
Figure S11. Structure characterization of Sn nanocrystal synthesized ex-situ.
Figure S12. Resolution of TEM image in thin-layer liquid cell.
Figure S13. Influence of chloride ions on the etching process.
Figure S14. The Kinetic Monte Carlo simulation on the corrosion of Sn with protection layer.
Figure S15. The crystal structure and in-plan direction of (020) and (011) of β-Sn.

Legends for movies Supplementary Video 1 to 8

Other Supplementary Materials for this manuscript include the following:

Supplementary Video 1 to 8

Materials and Methods

Materials

Materials: Ultra-thin carbon film (10 nm, 400 mesh) supported copper grids were purchased from Electron Microscopy Sciences (EMS). All commercially available chemicals including Tin chloride (99.995%), Sodium stannate hydrate (95%), Beryllium chunks (99.9%), Nickel(II) chloride (99.99%), $NaBH_4$ (>=96%), Poly(diallyldimethylammonium chloride) (PDDA) solution (20 wt% in H_2O, average Mw: 200,000-350,000) were received as used.

Liquid cell fabrication and in situ TEM experiment

We first treated two ultrathin (10 nm) TEM carbon grids with oxygen/argon plasma for 30 s to produce a hydrophilic surface for improved wetting of the highly polar aqueous solution (Figure S1). The liquid solution containing Sn nanocrystals was prepared by a simple redox reaction between Be metal and $SnCl_4$. 200 μL of 20 mg/mL $SnCl_4$ solution (a small amount of hydrochloric acid was added to prevent the hydrolysis of $SnCl_4$) was first dropped on the top of the Be metal chunk for 10 s. The liquid solution containing Sn@Ni_3Sn_4 was prepared by dropping the mixture solution of 200 μL of 20 mg/mL $SnCl_4$ and 1mg/mL $NiCl_2$ solution on the top of the Be metal chunk. Touching the liquid drop with a carbon-film-supported TEM grid allows the transfer of the nanocrystals into liquid. The droplet with nanocrystals was then sandwiched by another carbon-film TEM grid to form thin liquid pockets between them (Figure S2). The van der Waals force between the top and bottom carbon films contributed to sealing a small amount of solution in some pockets. The liquid cell was left in a homemade vacuum stage for 0.5 h to remove water that was not sealed in the pocket. Moreover, the drying process under a vacuum could prevent the nanocrystals from being oxidized. After that, the sample was loaded into the TEM for an in situ study.

Ex-situ synthesis of Sn@SnO_2 nanorods

The Sn@SnO_2 nanocrystals were synthesized based on a previously reported recipe[1]. Firstly, 0.1 g $NaBH_4$ and 0.5 mL poly(diallyldimethylammonium chloride) (PDDA) were dissolved in 60 ml deionized water. 30 mL of 3 mg/ml $SnCl_4$ solution was added dropwise to the above-mixed solution of $NaBH_4$ and PDDA. The resulting gray solid products were washed with deionized water and ethanol.

TEM characterization

The Thermo Fisher aberration-corrected TEM (ThemIS) at the Molecular Foundry (MF), Lawrence Berkeley National Laboratory (LBNL), was used for in situ observations and HAADF-STEM characterization. The microscope, operating voltage and electron beam intensity for each experiment are indicated in the corresponding movie captions. The materials characterization at low temperature was achieved using a Gatan cryo-holder.

Imaging processing

For image analysis of the etching process, custom Matlab scripts were used to extract the outline and quantify area change and facet velocity. Each frame of the TEM videos was filtered to enhance contrast, and then thresholded to yield the outline in a similar method to previously reported literature [2]. After obtaining the outline of each object, the areas of etching holes and facet etching rates were measured. Areas were measured using thresholding by inversing the contrast of the image. Facet etching rates were found by measuring the orthogonal distance from a line through the facet to a line through the initial facet. Distances in Figure 2 and Figure 3 represent the orthogonal distance of the facet from its initial position. During the imaging process of the in situ study, it is challenging to capture the initial corrosion process due to the limited viewing area under TEM. Therefore, recording only began when a corrosion event occurred in the field of view. Thus, time = 0.0 s in all TEM images is the moment that we started to capture the corrosion process and not the beginning of the corrosion reaction.

Calculation

An in-house atomistic kinetic Monte Carlo engine was developed according to the reference[3], adapted to the white tin viewing along [100]. The simulation temperature is 298 K, with a corrosion rate of 0.1 ML/s (monolayers per second). The bond energy of white tin is calculated from the Density Function Theory simulation to be 0.511 eV/bond. The diffusion barrier of the Sn atom on the Sn surface is calculated to be 0.214 eV.

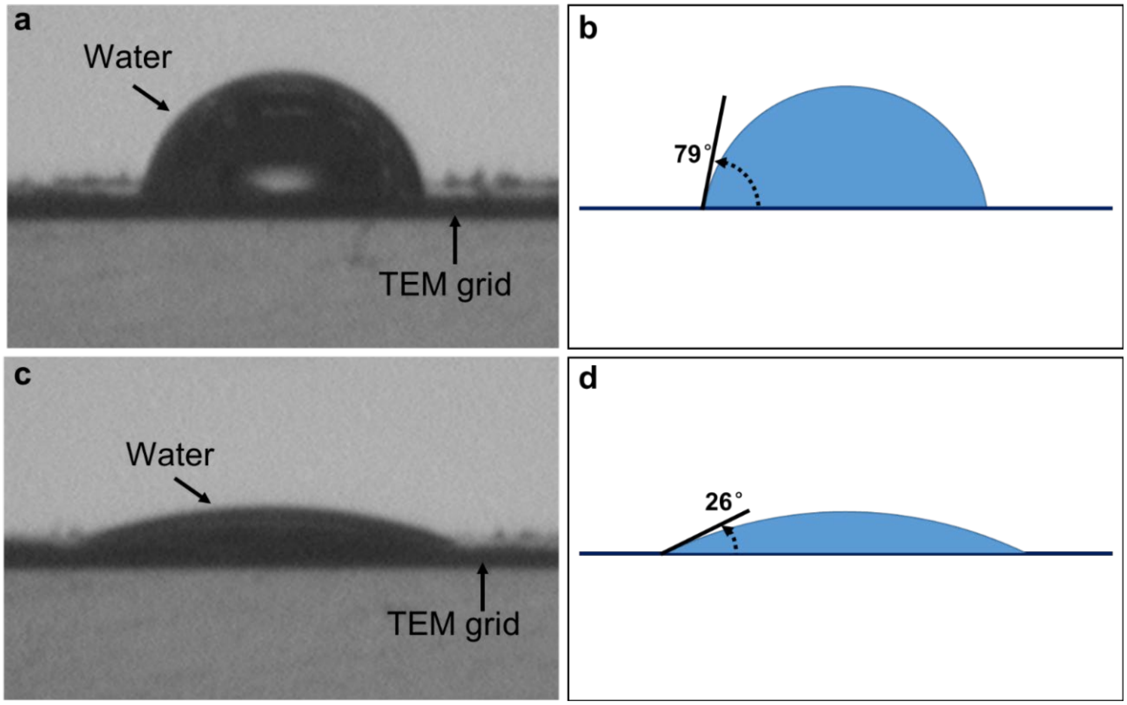

Figure S1. Surface classification based on contact angles. (a) Image and (b) contact angle measurement of a drop of water on a commercial TEM grid. (c) Image and (d) contact angle measurement of a drop of water on an oxygen-plasma-treated TEM grid. The contact angle changes from 79° to 26° after the plasma cleaning. These figures demonstrate the effect of oxygen plasma treatment on the surface properties of the TEM grid. The contact angle, which is a measure of how effectively the surface repels or attracts a liquid, decreases significantly after the plasma cleaning, indicating an increased hydrophilicity or wettability of the surface. This change in contact angle suggests that the plasma treatment has modified the surface chemistry and surface energy, leading to improved wetting and adhesion of the water droplet on the grid.

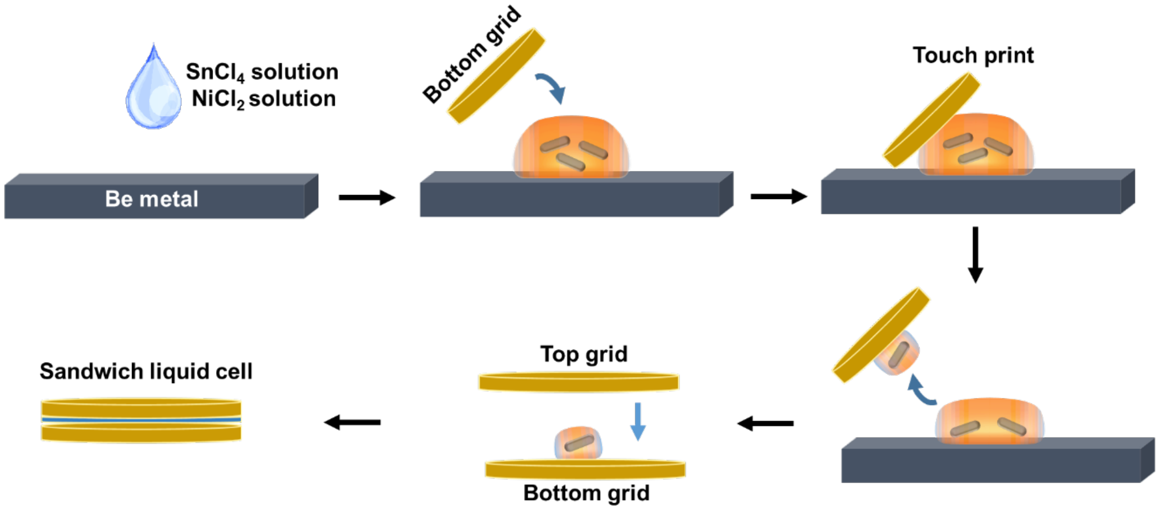

Figure S2. Schematic of representation of the set-up of the liquid-cell experiment. The $SnCl_4$ liquid droplet was dropped on the surface of the Be metal to produce Sn nanocrystals. Touching the liquid drop with a carbon-film-supported TEM grid allows the transfer of the nanocrystals into liquid. A top grid was assembled with the bottom grip to fabricate the sandwich liquid cell.

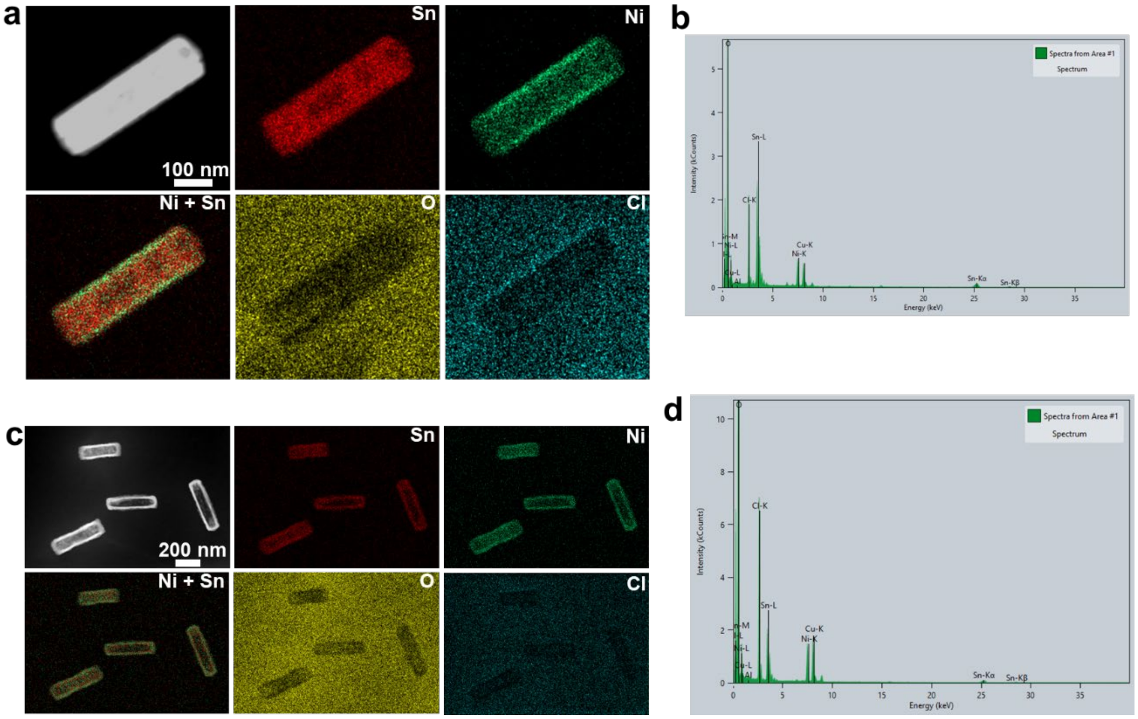

Figure S3. Morphology and composition characterization of Sn nanocrystals with nanocoating before and after corrosion. (a) HAADF-STEM image, EDS elements mapping of Sn, Ni, O and Cl, and (b) corresponding EDS spectrum of nanocrystals before corrosion. (c) HAADF-STEM image, EDS elements mapping of Sn, Ni, O and Cl, and (d) corresponding EDS spectrum of nanocrystals after corrosion.

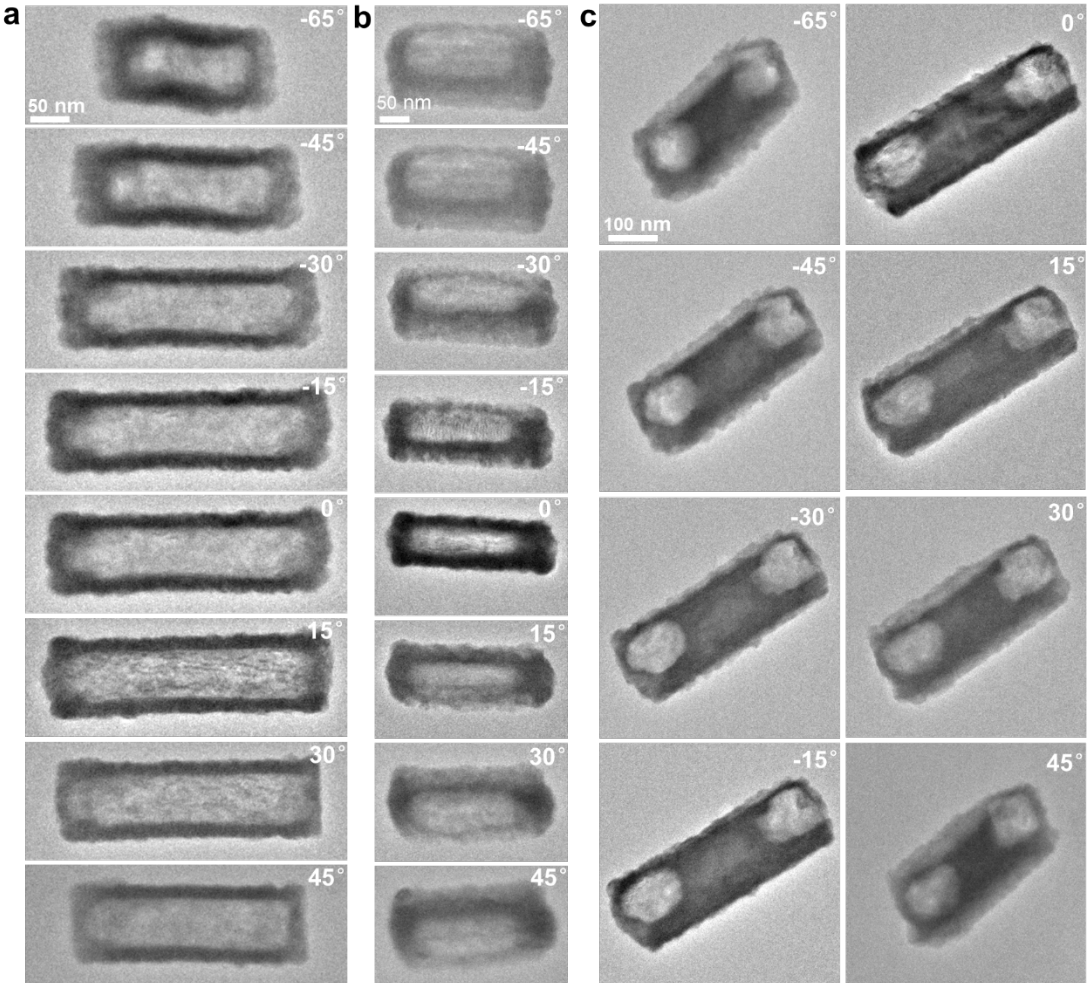

Figure S4. Electron tomography images of Sn nanocrystals. (a-c) TEM images of different particles at different tilting angles.

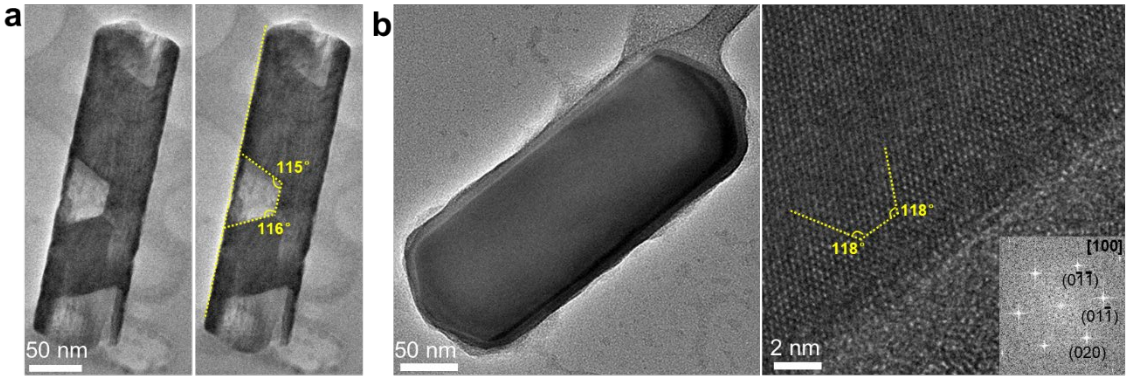

Figure S5. Facet determination according to the etched morphology. (a) Representative TEM images of the Sn intermediate during the pitting corrosion process. The angles between different facets are measured. (b) Low- and high-magnification TEM images of Sn nanorod.

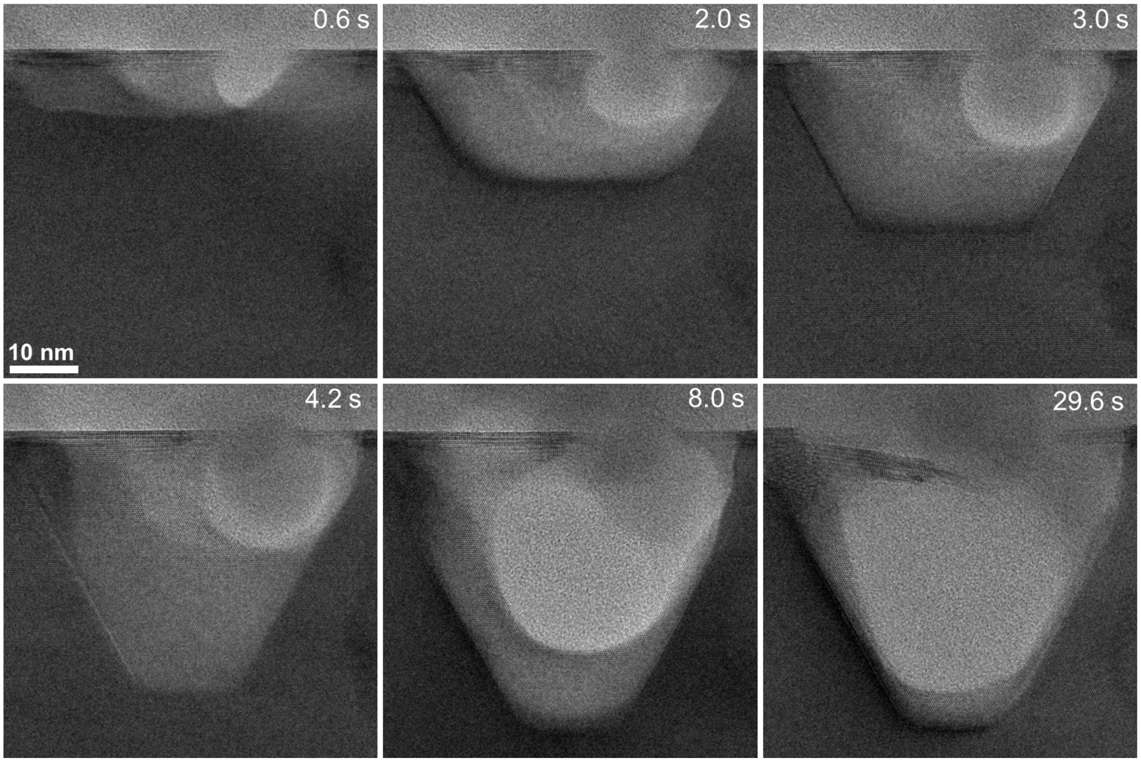

Figure S6. High-resolution real-time observation of the oxidative pitting corrosion of Sn@Ni_3Sn_4 nanocrystal in aqueous solution.

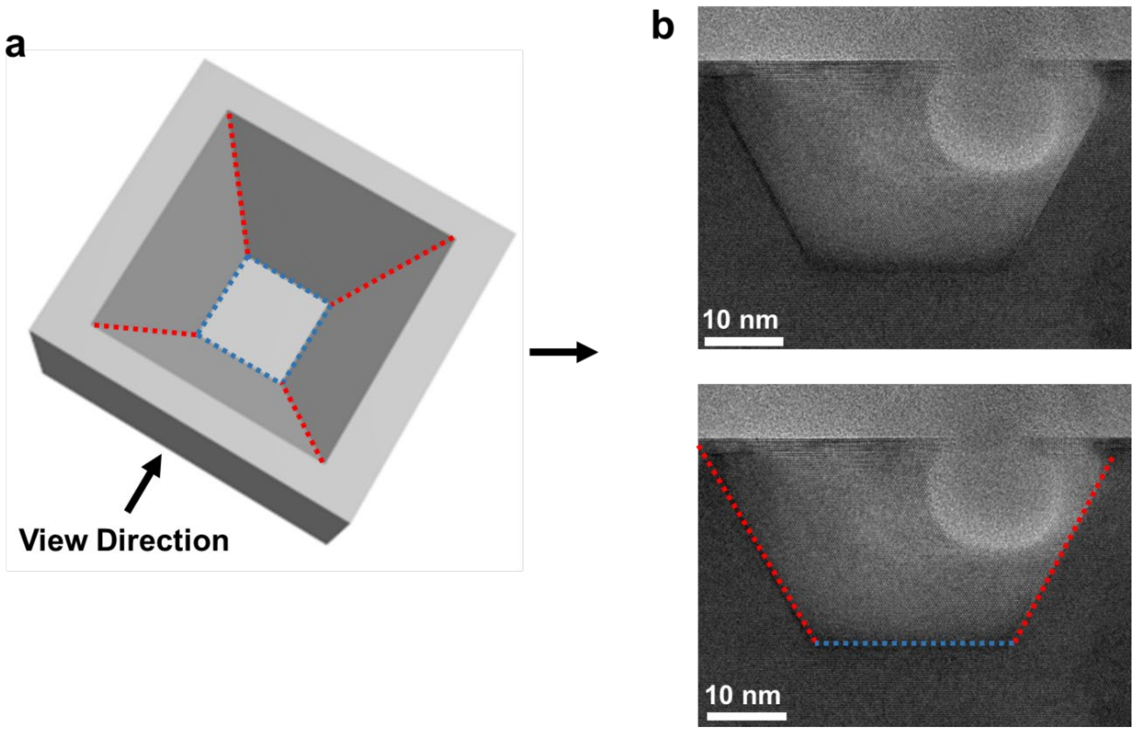

Figure S7. 3D model of rectangular prism nanocrystal viewing along [100] direction. (a) 3D model of the nanocrystal after pitting corrosion. An anisotropic etching creates a cavity with a trapezoidal cross-section. (b) The top image is the high-resolution TEM image of the Sn@Ni_3Sn_4 at 3.0 s from Supplementary Video 3. The bottom image is labeled with red and blue dash lines, which correspond to the projection image of the 3D model.

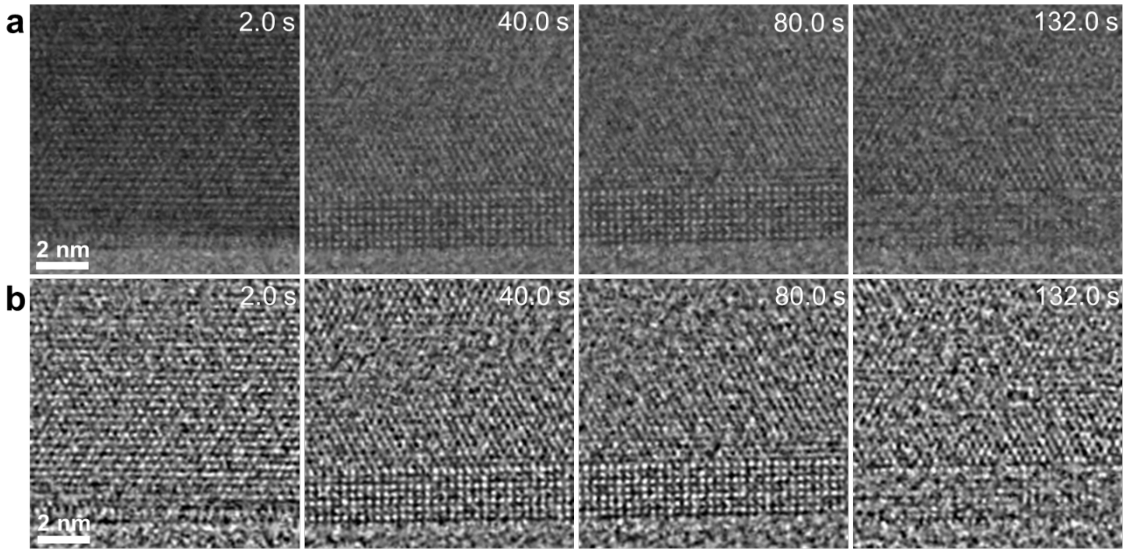

Figure S8. Sequential TEM images show that no corrosion reaction occurs where the protective layer is intact. (a) Selected original keyframes and (b) after band-pass filter treatment.

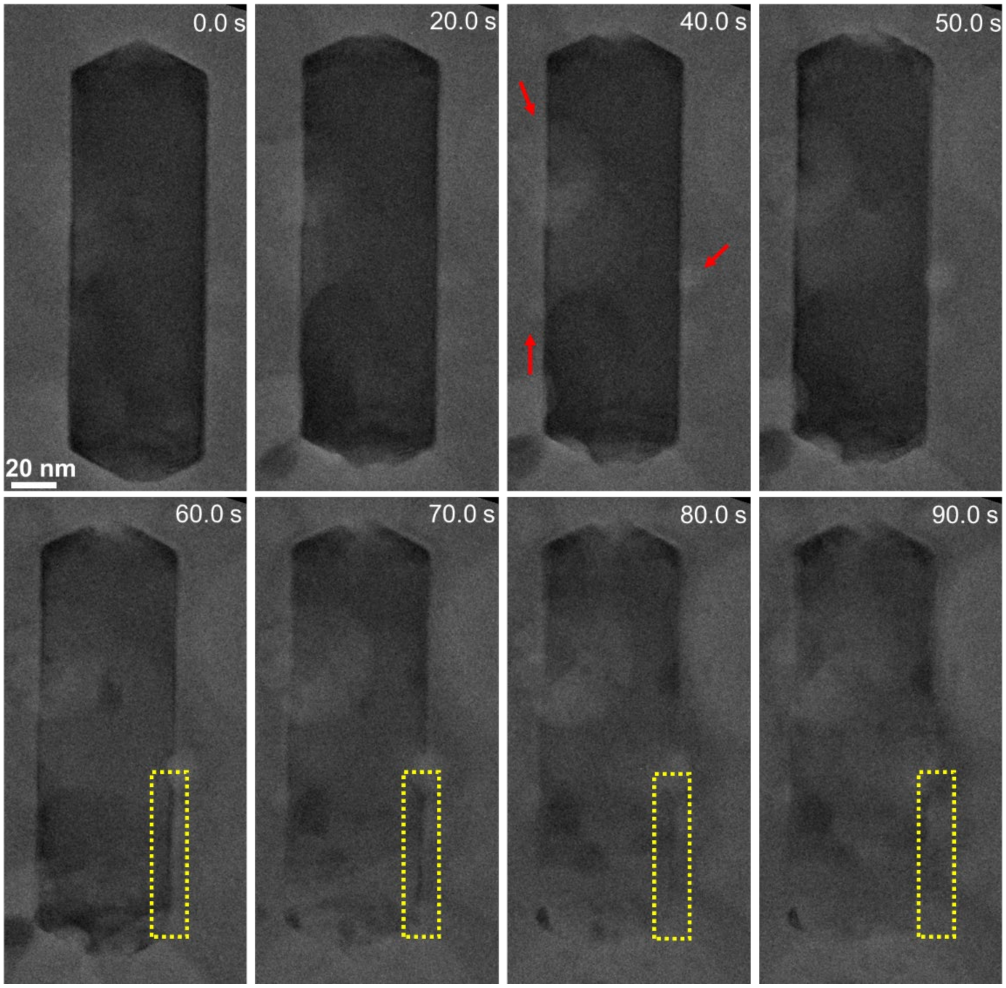

Figure S9. Sequential TEM images showing the corrosion process of Sn@Ni_3Sn_4 nanocrystal in aqueous solution.

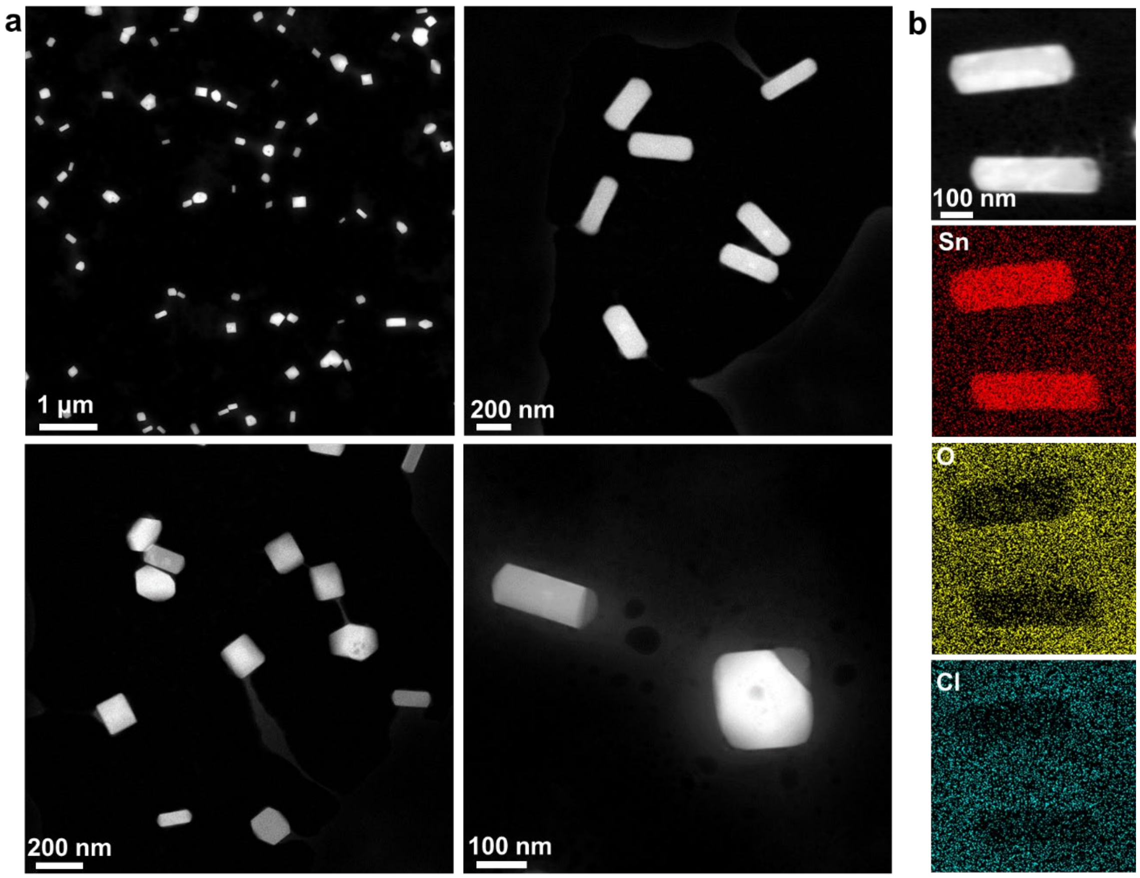

Figure S10. Structural morphology and EDS characterization of Sn nanocrystal within the liquid cell by cryo-EM. (a) HAADF-STEM images of Sn nanocrystal and (b) corresponding elements mapping of Sn, O, and Cl. Different morphologies of Sn nanocrystals are observed, and no oxidation layer is detected on the surface of Sn nanocrystal.

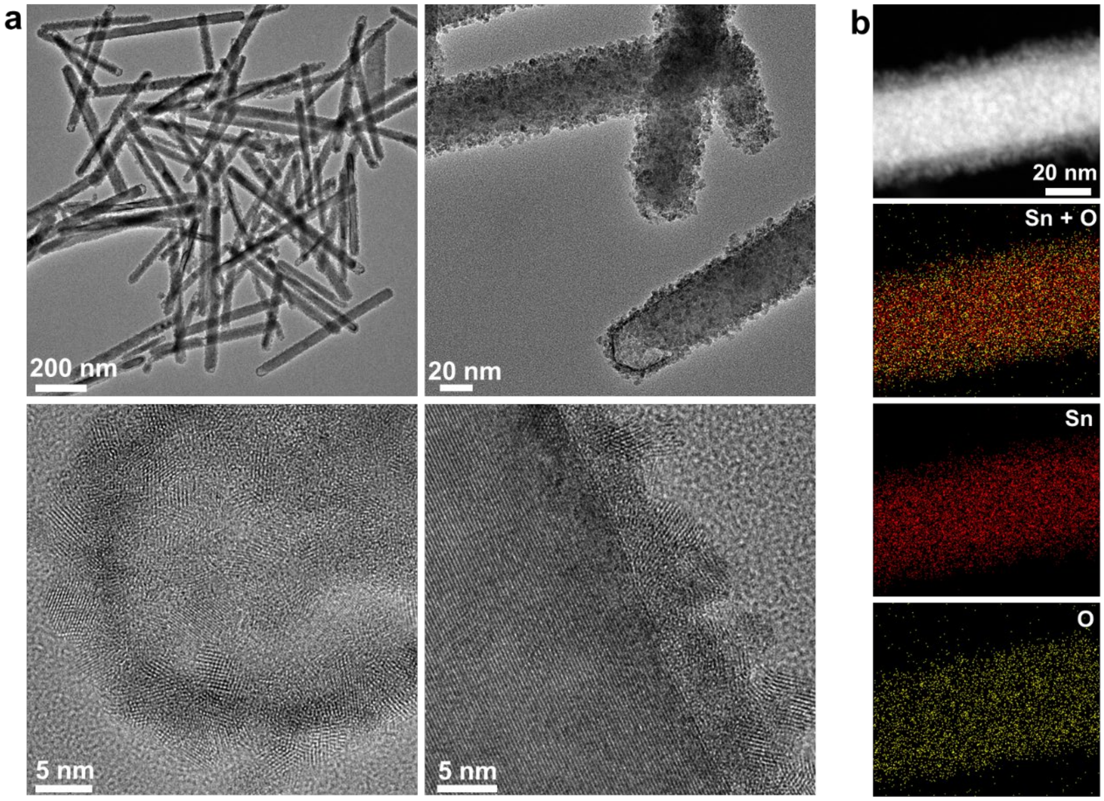

Figure S11. Characterization of Sn nanocrystal synthesized ex-situ. (a) Low- and high-magnification TEM images of Sn nanorods. The high-resolution image indicates the polycrystalline SnO_2 layer is formed on the surface of the Sn nanorod. (b) EDS elemental maps of Sn, O confirm the existence of a surface oxidation layer.

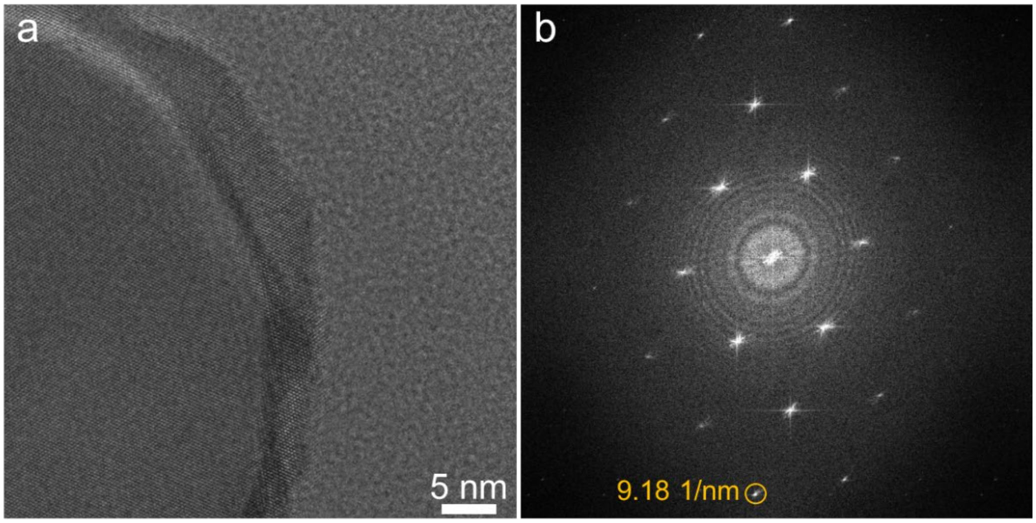

Figure S12. Resolution of TEM image in thin-layer liquid cell. (a) High-resolution TEM image of the Sn nanocrystal in the liquid cell, as previously shown in Figure 4b. (b) The corresponding Fourier transform of image a. The resolution was calculated to be 0.109 nm, based on the spot marked by the orange circle.

Inelastic scattering in thick liquid samples, lens instabilities and the intrinsic energy spread of the source all contribute to a spread in energy of the electrons that form the image. This energy spread reduces the resolution because of chromatic aberration (C_C). For water, and in the non-relativistic case, we can estimate for the TEM resolution in the water system based on the following equation[4, 5]:

$$d = 6 \times 10^{12} \alpha C_C \cdot t / E^2$$

where d is the TEM resolution, α is the objective aperture semi-angle, C_C is the chromatic aberration coefficient, t is thickness of liquid layer, and E is the beam energy.

In our system, when neglecting the windows and using the parameters α=10 mrad, C_C=2 mm, and E=300 keV, the thickness of the liquid layer is calculated to be 82 nm, considering a resolution of 0.109 nm from the representative high-resolution TEM image shown in Figure S12.

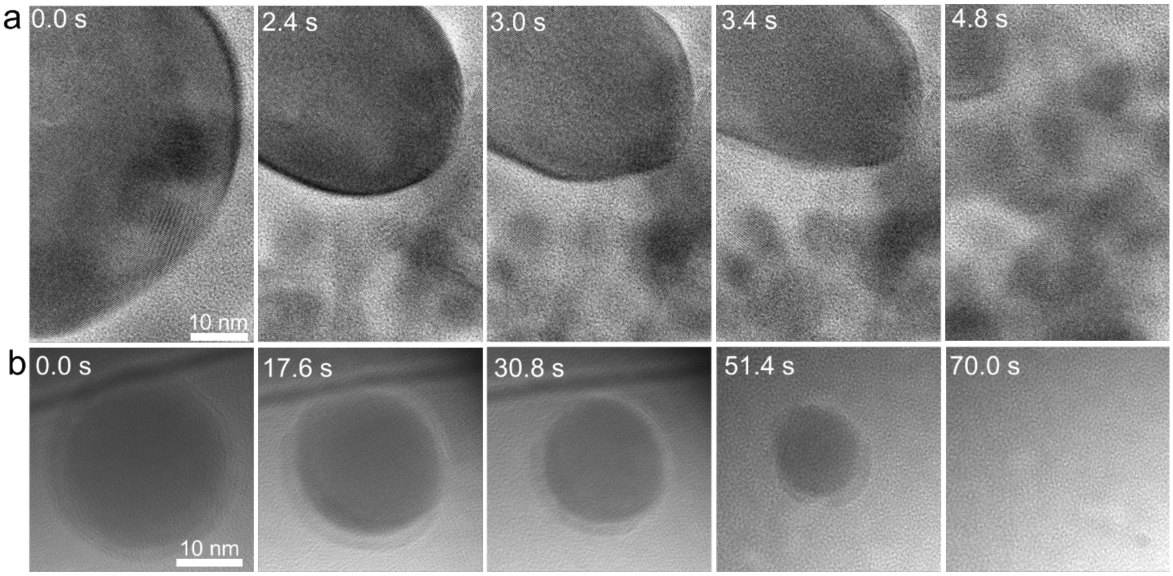

Figure S13. Influence of chloride ions on the etching process. (a) High-resolution TEM image series of the Sn nanocrystal: (a) in a solution with chloride ions and (b) in a solution without chloride ions. The dose rate used for imaging in a and b is around 7470 $e^{-}\cdot Å^{-2}\cdot s^{-1}$. In order to eliminate the influence of chloride ions, Sn particles were synthesized by reducing Sodium Stannate inside the liquid cell. This synthesis method resulted in the formation of a quasi-liquid layer on the surface of the Sn nanocrystal[6]. Upon examining the TEM image series in Figure S13, it can be observed that the dissolution kinetics of the Sn particles is significantly slower in the solution without chloride ions compared to the solution containing chloride ions. By comparing the behavior of Sn in aqueous solutions with chloride ions and without chloride ions, we can observe that chloride ions have the potential to accelerate the corrosion reaction.

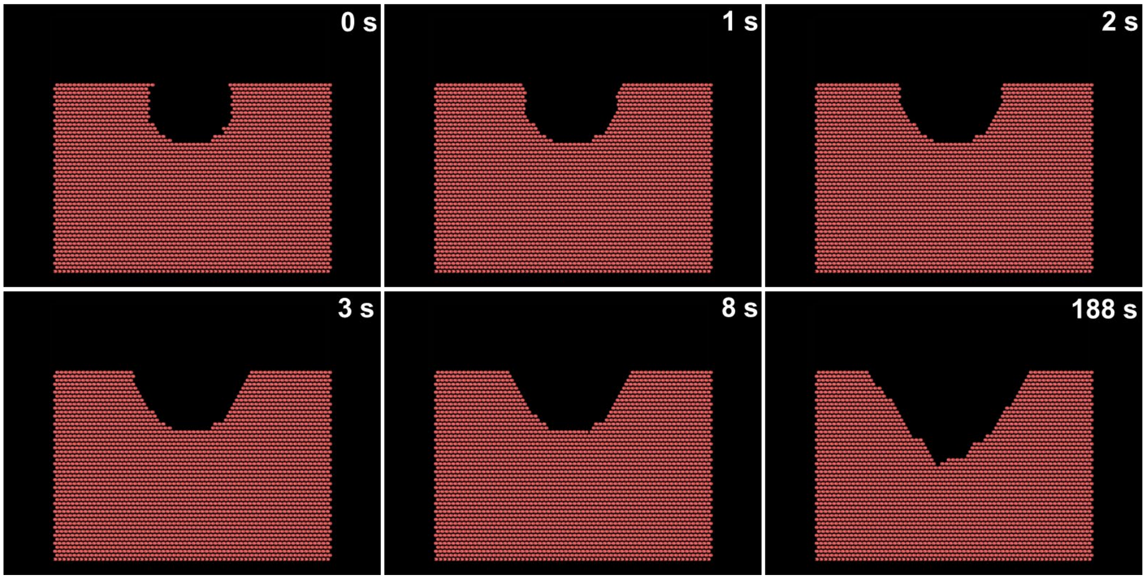

Figure S14. The Kinetic Monte Carlo simulation on the corrosion of Sn with protection layer. Snapshots of the trajectory of Sn nanocrystals after removing Sn atoms from the pitting region.

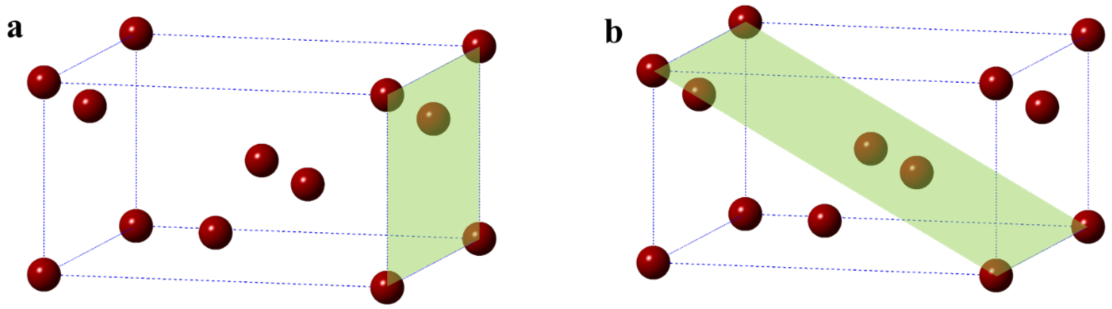

Figure S15. The crystal structure and in-plane direction of (020) and (011) of β-Sn. (a) The united cell of β-Sn and the in-plane direction of (020). (b) The united cell of β-Sn and the in-plane direction of (011).

Supplementary Movie Captions

Supplementary Video 1

3D reconstruction of a single nanocrystal

Supplementary Video 2

In-situ liquid cell TEM movie showing the pitting corrosion process of Sn@Ni_3Sn_4 nanocrystals. The movie was recorded using ThemIS under 300 keV with a dose rate of 1210 $e^{-}\cdot Å^{-2}\cdot s^{-1}$.

Supplementary Video 3

In-situ liquid cell TEM movie showing the pitting corrosion process of Sn@Ni_3Sn_4 nanocrystals. The movie was recorded using ThemIS under 300 keV with a dose rate of 6105 $e^{-}\cdot Å^{-2}\cdot s^{-1}$.

Supplementary Video 4

In-situ liquid cell TEM movie S3 after band-pass filter to show the initial pitting corrosion.

Supplementary Video 5

In-situ liquid cell TEM movie showing the galvanic corrosion process, and the influence of bubbles on corrosion behaviors at low magnification. The movie was recorded using ThemIS under 300 keV with a dose rate of 751 $e^{-}\cdot Å^{-2}\cdot s^{-1}$.

Supplementary Video 6

In-situ liquid cell TEM movie showing the fast corrosion kinetics of metallic Sn nanocrystals in the thick liquid layer at high magnification. The movie was recorded using ThemIS under 300 keV with a dose rate of 7470 $e^{-}\cdot Å^{-2}\cdot s^{-1}$.

Supplementary Video 7

In-situ liquid cell TEM movie showing the “creep-like” etching process and diffusion layer during the uniform corrosion process in the thin liquid layer at high magnification. The movie was recorded using ThemIS under 300 keV with a dose rate of 12000 $e^{-}\cdot Å^{-2}\cdot s^{-1}$.

Supplementary Video 8

The Kinetic Monte Carlo simulation on the corrosion of Sn with protection layer.

References

1. Du, N.; Zhang, H.; Chen, B.; Ma, X.; Yang, D., One-pot, large-scale synthesis of SnO2nanotubes at room temperature. *Chem. Commun.* **2008,** (26), 3028-3030.
2. Hauwiller, M. R.; Frechette, L. B.; Jones, M. R.; Ondry, J. C.; Rotskoff, G. M.; Geissler, P.; Alivisatos, A. P., Unraveling kinetically-driven mechanisms of gold nanocrystal shape transformations using graphene liquid cell electron microscopy. *Nano Lett.* **2018,** *18* (9), 5731-5737.
3. Nie, Y.; Liang, C.; Cha, P.-R.; Colombo, L.; Wallace, R. M.; Cho, K., A kinetic Monte Carlo simulation method of van der Waals epitaxy for atomistic nucleation-growth processes of transition metal dichalcogenides. *Sci. Rep-Uk* **2017,** *7* (1), 1-13.
4. de Jonge, N.; Ross, F. M., Electron microscopy of specimens in liquid. *Nat. Nanotechnol.* **2011,** *6* (11), 695-704.
5. de Jonge, N.; Houben, L.; Dunin-Borkowski, R. E.; Ross, F. M., Resolution and aberration correction in liquid cell transmission electron microscopy. *Nature Reviews Materials* **2018,** *4* (1), 61-78.
6. Peng, X.; Zhu, F.-C.; Jiang, Y.-H.; Sun, J.-J.; Xiao, L.-P.; Zhou, S.; Bustillo, K. C.; Lin, L.-H.; Cheng, J.; Li, J.-F., Identification of a quasi-liquid phase at solid–liquid interface. *Nat. Commun.* **2022,** *13* (1), 3601.
